# The relationship between prenatal psychological stress and placental abruption in Japan, The Japan Environment and Children’s Study (JECS)

**DOI:** 10.1371/journal.pone.0219379

**Published:** 2019-07-08

**Authors:** Yasuyuki Kawanishi, Eiji Yoshioka, Yasuaki Saijo, Toshihiro Itoh, Toshinobu Miyamoto, Kazuo Sengoku, Yoshiya Ito, Sachiko Ito, Chihiro Miyashita, Atsuko Araki, Toshiaki Endo, Kazutoshi Cho, Hisanori Minakami, Reiko Kishi, for the Japan Environment and Children’s Study Group

**Affiliations:** 1 Department of Social Medicine, Asahikawa Medical University, Asahikawa, Hokkaido, Japan; 2 Center for Baby Science, Doshisha University, Kizugawa, Kyoto, Japan; 3 Nagaoka Healthcare Center, Nagaokakyo, Kyoto, Japan; 4 Laboratory of Public Health, Department of Nursing, Asahikawa Medical University, Asahikawa, Hokkaido, Japan; 5 Department of Obstetrics and Gynecology, Asahikawa Medical University, Asahikawa, Hokkaido, Japan; 6 Faculty of Nursing, Japanese Red Cross Hokkaido College of Nursing, Kitami, Hokkaido, Japan; 7 Center for Environmental and Health Sciences, Hokkaido University, Sapporo, Hokkaido, Japan; 8 Department of Obstetrics and Gynecology, Sapporo Medical University, Sapporo, Hokkaido, Japan; 9 Center for Perinatal Medicine, Hokkaido University Hospital, Sapporo, Hokkaido, Japan; Chiba Daigaku, JAPAN

## Abstract

**Background:**

Prenatal psychological stress may increase the risk of placental abruption (PA). This study aimed to clarify the effects of psychological distress during pregnancy and exposure to stressful life events in the year before or during pregnancy on the occurrence of PA in Japanese women.

**Methods:**

Using a nationwide prospective birth cohort study, we obtained data from 103,099 women between January 2011 and March 2014. Information on exposure to 14 stressful life events and psychological distress (Kessler 6 scale) was collected using a self-administered questionnaire during pregnancy. Clinical diagnoses of PA were obtained from medical records. A total of 80,799 women with singleton births were analyzed using logistic regression models that adjusted for possible confounders.

**Results:**

PA was diagnosed in 335 (0.4%) women. There was no significant difference in the Kessler 6 score during pregnancy between the PA group and non-PA group. Exposure to the death of a child in the year before or during pregnancy was significantly associated with PA in multigravid women (adjusted odds ratio [aOR] 3.57; 95% confidence interval [CI] 1.50–8.34). A spouse’s loss of employment was significantly associated with PA in parous women (aOR 3.25; 95% CI 1.40–7.56).

**Conclusions:**

This study identified the possible effects of exposure to the death of a child on PA occurrence that adjusted for important confounding factors.

## Introduction

Placental abruption (PA) is a severe condition involving the partial or complete separation of a normally implanted placenta before delivery, and occurs in 0.4–1% of all pregnancies [1, 2]. This condition is one of the most important causes of maternal morbidity and perinatal mortality [1–3]. Although its detailed pathophysiology remains unclear, defective placentation and subsequent placental infarctions have been shown to be contributing factors for PA [4, 5].

Previous studies have reported that psychologically stressed pregnant women are at increased risk of intrauterine growth restriction (IUGR) [6, 7], pre-eclampsia [8], and PA [9, 10]. In a case-control study of several hundred pregnant women in Peru, de Paz et al. identified prenatal depressive, anxiety, and stress symptoms as independent risk factors for PA [10]. Laszlo et al. conducted a prospective cohort study of singleton births in Sweden and Denmark, and reported that the death of a child the year before or during pregnancy was associated with a 54% increase in the odds of PA occurrence [9]. To our knowledge, only these two studies have reported an association between maternal psychological stress and PA. However, one was a retrospective study and the other could not adjust for potentially important confounding factors such as smoking status and hypertensive disorders of pregnancy.

The purpose of this study was to clarify the effects of psychological distress during pregnancy and exposure to stressful life events in the year before or during pregnancy on the occurrence of PA with adjustment for important confounding factors using Japan’s largest prospective birth cohort study.

## Methods

### Study participants

The Japan Environment and Children’s Study (JECS) is a prospective birth cohort study that involves participants located throughout Japan [11–13]. Women in the early stages of pregnancy are recruited into the JECS with written informed consent.

### Ethical issues

The JECS protocol was approved by the Institutional Review Board for Epidemiological Studies at the Ministry of the Environment, Japan, and the ethics committees of all participating institutions. These institutions include the National Institute for Environmental Studies (which leads the JECS), the National Center for Child Health and Development, Hokkaido University, Sapporo Medical University, Asahikawa Medical College, Japanese Red Cross Hokkaido College of Nursing, Tohoku University, Fukushima Medical University, Chiba University, Yokohama City University, University of Yamanashi, Shinshu University; University of Toyama, Nagoya City University, Kyoto University, Doshisha University, Osaka University, Osaka Medical Center and Research Institute for Maternal and Child Health, Hyogo College of Medicine, Tottori University, Kochi University, University of Occupational and Environmental Health, Kyushu University, Kumamoto University, University of Miyazaki, and the University of the Ryukyus. The JECS was conducted in accordance with the Helsinki Declaration and other regulations and guidelines stipulated by the Japanese government.

### Exposure variable

The exposure variables included the experience of a stressful life event and psychological distress. A self-administered questionnaire about stressful life events was distributed to each participant during their second/third trimester (T2 period). Participants were asked “Did you experience any of the following stressful events during the past year?”. If the answer was “Yes”, the participant selected an option(s) from the following list: “Death of a parent”, “Death of a spouse”, “Death of a child”, “Illness of a parent”, “Illness/injury of a spouse”, “Illness/injury of a child”, “Spouse's loss of employment”, “Loss of employment”, “Death of a close friend”, “Taking out a major loan”, “Change in family structure (e.g., starting living together with grandparents)”, “Divorce”, “Moving house”, and “Having marital problems”.

Psychological distress was examined using the Kessler 6 scale (K6), which is one of the most widely employed as an indicator of mental disorders. [14] Because of its shortness, K6 has a great advantage than other well-known scales. The reliability and validity of the original version have been repeatedly confirmed. [15, 16] The Japanese version of the K6 was developed using the standard back-translation method [17], and has been previously validated [18]. The K6 consists of six items, each of which has five possible responses and corresponding scores (0–4). A higher total score (range 0–24) indicates a more psychologically distressed state, and Kessler et al [16]. showed those with K6 scores of ≥ 13 as having psychological distress. We distributed the questionnaire to measure K6 twice during pregnancy: once during their first trimester (T1 period) and a second time during their T2 period.

### Outcome variable

The outcome variable of this study was clinically diagnosed PA based on the relevant International Classification of Diseases, 10th Revision code (O45). The clinical diagnosis was ascertained from medical records by physicians, midwives/nurses, and/or research coordinators after delivery [12]. Participants who developed PA were designated the PA group, and the remaining participants were designated the non-PA group.

### Covariates

We collected information on potential confounding factors of PA [2, 19]. Using a self-administered questionnaire sent to the participants during the T1 period, we collected data on marital status, smoking status, employment status, history of uterine fibroids, and history of hypertensive disorders of pregnancy. Using another self-administered questionnaire sent during the T2 period, we also collected data on alcohol consumption during pregnancy and maternal education level. The following information was collected from medical records: maternal age at delivery, parity, use of infertility treatment, pre-pregnancy body mass index (BMI), chronic hypertension, diabetes mellitus, psychiatric disorder, hypothyroidism, uterine deformity, history of cesarean section, history of spontaneous abortion, history of stillbirth, history of PA, use of iron supplements during pregnancy, intrauterine infection, premature rupture of membranes, oligohydramnios, polyhydramnios, placenta previa, IUGR, gender of offspring, and hypertensive disorders of pregnancy. Of the above, the following disorders were clinically diagnosed based on the relevant International Classification of Diseases, 10th Revision code; premature rupture of membranes (O42), IUGR (P05), hypertensive disorders of pregnancy (O13, O14), gestational diabetes (O24.4, O24.9), placenta previa (O42), polyhydramnios (O40) and oligohydramnios (O41).

### Statistical analysis

Analyses were performed using IBM SPSS Statistics 23.0 and 24.0 for Windows (SPSS Inc., Chicago, IL, USA). First, we compared the stressful life events occurrence and psychological distress status between the PA group and the non-PA group. These were calculated and presented as numbers and percentages or mean and standard deviations, where applicable. Fisher’s exact test was performed to assess categorical variables and Mann-Whitney *U* test was performed to assess continuous variables. Next, we compared the baseline characteristics for the “death of a child” exposure that had shown a significant association in the analysis.

Adjusted odds ratios (aORs) with 95% confidence intervals (CIs) for PA were estimated for each stressful life event and K6 score using multivariable logistic regression models. We conducted adjustments for maternal age at delivery, parity, marital status, smoking status, alcohol consumption, use of infertility treatment, maternal education level, pre-pregnancy BMI, employment status, chronic hypertension, diabetes mellitus, psychiatric disorder, hypothyroidism, uterine deformity, history of uterine fibroids, history of cesarean section, history of spontaneous abortion, history of stillbirth, history of PA, history of hypertensive disorders of pregnancy, use of iron supplements during pregnancy, intrauterine infection, premature rupture of membranes, oligohydramnios, polyhydramnios, placenta previa, IUGR, gender of offspring, and hypertensive disorders of pregnancy. These covariates were selected due to their previous identification as potential confounders of PA [2, 19], and were included in analysis using the forced entry method. For the following stressful events, the sample was restricted to women who had experienced pregnancy at least once before the index pregnancy: for the “death of a child” exposure, only multigravid women (n = 55,475) were included in analysis; for the “illness/injury of a child” exposure, only parous women (n = 46,026) were included in analysis.

Subsequently, we performed stratified analyses according to parity for three reasons. The first reason was to confirm the influence of the “death of a child” exposure on PA in parous women. The second reason was that childcare is known to be stressful for mothers [20], and the influence of this stressful life event exposure may be influenced by parity. Third, the PA risk factors may differ between nulliparous and parous women [21]. We performed multivariable logistic regression analysis stratified by parity for two stressful life events (“death of a child” and “spouse’s loss of employment”) that had shown a significant or a marginally significant association (*P*<0.1) in the earlier multivariable analyses.

We also compared the perinatal outcomes, such as gestational age, birth weight, stillbirth, mode of delivery, Apgar score, pH value of umbilical arterial blood, maternal blood transfusion, maternal care in the intensive care unit (ICU), and maternal death, for the purpose of confirming the clinical severity of PA. Additionally, we compared the baseline characteristics of the PA occurrence. Finally, we compared the final analyzed participants (n = 80,799) and the excluded participants for the reason of missing variables (n = 15,463). Statistical significance was set at *P*<0.05 for all analyses.

## Results

**Fig 1:**
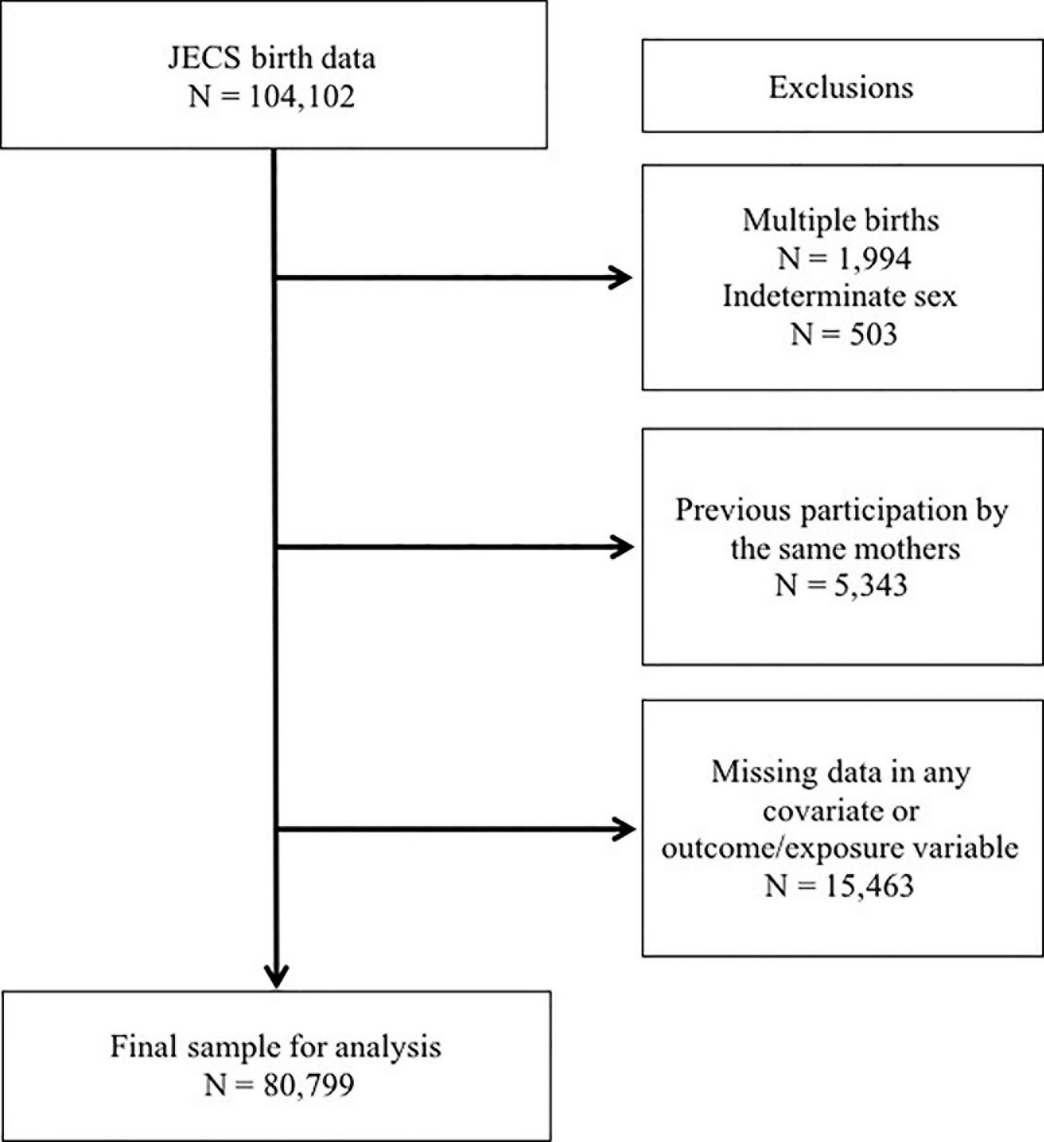
Study sample selection flow diagram.

There were 103,099 participants between January 2011 and March 2014, and the study selection flow diagram is shown in Fig 1. Of the 104,102 births (JECS data code: jecs-ag-20160424 and revised in October 2016), we excluded multiple births (n = 1,994); babies of indeterminate sex (n = 503); previous participation in this study by the same mothers (n = 5,343); and missing data in any covariate, outcome variable, or exposure variable (n = 15,463). The final study sample comprised 80,799 women.

Of the 80,799 women in the study sample, 335 (0.4%) developed PA. There were no significant differences in K6 score in both the T1 and T2 periods between the PA and non- PA groups though, exposure to the “death of a child” showed the significant difference (Table 1).

**Table 1:** Participant stressful life event occurrence and psychological distress status according to clinical diagnosis of placental abruption.

	No abruption	Abruption	
Variables	(n = 80,464)	(n = 335)	P-value
Stressful life event			
Death of a parent	1,378 (1.7)	7 (2.1)	0.52
Death of a spouse	66 (0.1)	0 (0)	1.00
Death of a child^a^	373 (0.7)	6 (2.6)	0.005
Illness or injury of a parent	4,381 (5.4)	24 (7.2)	0.18
Illness or injury of a spouse	2407 (3.0)	7 (2.1)	0.42
Illness or injury of a child^b^	5,110 (11.1)	22 (12.1)	0.65
Spouse's Loss of employment	786 (1.0)	7 (2.1)	0.05
Loss of employment	952 (1.2)	6 (1.8)	0.30
Death of a close friend	859 (1.1)	5 (1.5)	0.42
Taking out a major loan	679 (0.8)	5 (1.5)	0.21
Change in family structure	3487 (4.3)	8 (2.4)	0.08
Divorce	247 (0.3)	1 (0.3)	1.00
Moving house	8,326 (10.3)	29 (8.7)	0.37
Having marital problems	8,020 (10.0)	34 (10.1)	0.87
K6 score in T1 period	3.7 (3.8)	3.6 (3.9)	0.77
K6 score in T2 period	3.5 (3.7)	3.6 (3.9)	0.44

NA, not available; K6, Kessler 6 scale; T1, first trimester; T2, second/third trimester.

^a^Gravidity ≥2 only (n = 55,475; no abruption n = 55,247, abruption n = 228).

^b^Parity ≥1 only (n = 46,026; no abruption n = 45,844, abruption n = 182).

Data are presented as n (%) or mean (standard deviation).

P-values based on a Fisher’s exact test (for categorical variables) or a Mann-Whitney U test (for continuous variables).

The participant characteristics according to the exposure or non-exposure to the death of a child are shown in Table 2. When compared with the non-exposed group, the proportions of participants significantly differ in parity, smoking status, history of uterine fibroids, history of cesarean section, history of spontaneous abortion, history of stillbirth, history of PA, oligohydramnios and K6 score both in T1 and T2 compared with the “death of a child” exposed group among multigravid women.

**Table 2:** Participant characteristics according to maternal exposure or non-exposure to the death of a child one year before or during pregnancy in multigravid women.

	Death of a child		
Characteristics	No (n = 55,096)	Yes (n = 379)	P-value
Age (years) at delivery			0.08
<20	157 (0.3)	1 (0.3)	
20–24	3,268 (5.9)	32 (8.4)	
25–29	13,207 (24.0)	105 (27.7)	
30–34	20,905 (37.9)	130 (34.3)	
≥35	17,559 (31.9)	111 (29.3)	
Parity			<0.001
0	9,299 (16.9)	153 (40.4)	
1	30,017 (54.5)	138 (36.4)	
2	12,504 (22.7)	62 (16.4)	
≥3	3,276 (5.9)	26 (6.9)	
Marital status			0.06
Married (including common-law marriage)	53,549 (97.2)	362 (95.5)	
Single (Never married, Divorced, Widowed)	1,547 (2.8)	17 (4.5)	
Smoking status			0.01
Never	30,294 (55.0)	223 (58.8)	
Quit before awareness of current pregnancy	14,478 (26.3)	80 (21.1)	
Quit after awareness of current pregnancy	7,261 (13.2)	63 (16.6)	
Currently smoking	3,063 (5.6)	13 (3.4)	
Alcohol consumption during pregnancy			0.20
Never	18,835 (34.2)	129 (34.0)	
Quit before awareness of current pregnancy	10,098 (18.3)	73 (19.3)	
Quit after awareness of current pregnancy	24,217 (44.0)	171 (45.1)	
Currently drinking	1,946 (3.5)	6 (1.6)	
Infertility treatment			0.17
None (spontaneous pregnancy)	52,146 (94.6)	351 (92.6)	
Ovulation induction, AIH	1,625 (2.9)	16 (4.2)	
ART	1,325 (2.4)	12 (3.2)	
Maternal education level			0.94
Junior high school	2,922 (5.3)	19 (5.0)	
High school	18,023 (32.7)	119 (31.4)	
Technical junior college, Technical/vocational college, Associate degree	23,240 (42.2)	164 (43.3)	
Bachelor's degree, Graduate degree (Master's/Doctor's)	10,911 (19.8)	77 (20.3)	
Pre-pregnancy BMI (kg/m^2^)			0.95
<18.5	8,470 (15.4)	58 (15.3)	
18.5–24.9	40,327 (73.2)	280 (73.9)	
≥25.0	6,299 (11.4)	41 (10.8)	
Current employment status			0.85
Permanent full-time employee, Self-employed	17,630 (32.0)	128 (33.8)	
Temporary full-time employee, Part-time employee	12,940 (23.5)	84 (22.2)	
Full-time homemaker or leave of absence	22,751 (41.3)	154 (40.6)	
Unemployed, Other	1,775 (3.2)	13 (3.4)	
Chronic hypertension	837 (1.5)	3 (0.8)	0.39
Prepregnant diabetes	564 (1.0)	7 (1.8)	0.12
Psychiatric disease	368 (0.7)	4 (1.1)	0.33
Hypothyroidism	362 (0.7)	5 (1.3)	0.11
Uterine deformity	21 (0.0)	0 (0)	1.00
History of uterine fibroids	3,252 (5.9)	36 (9.5)	0.004
History of cesarean section	6,942 (12.6)	33 (8.7)	0.03
History of spontaneous abortion	15,193 (27.6)	218 (57.5)	<0.001
History of stillbirth	478 (0.9)	56 (14.8)	<0.001
History of placental abruption	169 (0.3)	5 (1.3)	0.007
History of pregnancy-induced hypertension	1,494 (2.7)	7 (1.8)	0.42
Use of iron supplements during pregnancy	24,336 (44.2)	175 (46.2)	0.43
Intrauterine infection	275 (0.5)	1 (0.3)	1.00
Premature rupture of membranes	3,751 (6.8)	30 (7.9)	0.42
Oligohydramnios	584 (1.1)	9 (2.4)	0.02
Polyhydramnios	217 (0.4)	4 (1.1)	0.07
Placenta previa	382 (0.7)	5 (1.3)	0.20
Intrauterine growth restriction	991 (1.8)	11 (2.9)	0.12
Gender of offspring (male)	38,253 (51.3)	198 (52.2)	0.72
Hypertensive disorders of pregnancy			0.68
None	53,642 (97.4)	367 (96.8)	
Mild	999 (1.8)	8 (2.1)	
Severe	455 (0.8)	4 (1.1)	
K6 score in T1 period	3.6 (3.8)	4.6 (4.5)	<0.001
K6 score in T2 period	3.4 (3.7)	4.2 (4.1)	<0.001
Placental abruption	222 (0.4)	6 (1.6)	0.005

AIH, artificial insemination by sperm from husband; ART, assisted reproduction technology; BMI, body mass index; K6, Kessler 6 scale; T1, first trimester; T2, second/third trimester.

Data are presented as n (%) or mean (standard deviation).

P-values based on a Fisher’s exact test (for categorical variables) or a Mann-Whitney U test (for continuous variables).

As shown in Table 3, the aOR for PA occurrence was higher in the "death of a child" exposed group compared with the non-exposed group in multigravid women (adjusted OR 3.54; 95% CI 1.50–8.34). The aOR was numerically higher in the "spouse’s loss of employment" exposed group, but this was not statistically significant (aOR 2.08; 95% CI 0.97–4.49).

**Table 3:** Adjusted odds ratio of placental abruption occurrence for each stressful life event and psychological distress status.

Variables	Adjusted ^c^ OR	(95% CI)	P-value
Stressful life event			
Death of a parent	1.11	(0.52–2.37)	0.79
Death of a spouse	NA	(NA)	NA
Death of a child^a^	3.54	(1.50–8.34)	0.004
Illness or injury of a parent	1.27	(0.83–1.95)	0.26
Illness or injury of a spouse	0.62	(0.29–1.33)	0.22
Illness or injury of a child^b^	1.10	(0.70–1.74)	0.68
Spouse's Loss of employment	2.08	(0.97–4.49)	0.061
Loss of employment	1.39	(0.61–3.18)	0.43
Death of a close friend	1.34	(0.55–3.27)	0.52
Taking out a major loan	1.70	(0.69–4.21)	0.25
Change in family structure	0.55	(0.27–1.12)	0.10
Divorce	1.14	(0.16–8.35)	0.90
Moving house	0.89	(0.60–1.32)	0.57
Having marital problems	1.05	(0.72–1.51)	0.81
K6 score in T1 period	1.00	(0.97–1.03)	0.95
K6 score in T2 period	1.01 ^d^	(0.98–1.04)	0.57

OR, odds ratio; CI, confidence intervals; NA, not available.

^a^Gravidity ≥2 only (n = 55,475).

^b^Parity ≥1 only (n = 46,026).

^c^Adjusted for maternal age at delivery, parity, marital status, smoking status, alcohol consumption, use of infertility treatment, maternal education level, pre-pregnancy body mass index, employment status, chronic hypertension, diabetes mellitus, psychiatric disorder, hypothyroidism, uterine deformity, history of uterine fibroids, history of cesarean section, history of spontaneous abortion, history of stillbirth, history of placental abruption, history of pregnancy-induced hypertension, use of iron supplements during pregnancy, intrauterine infection, premature rupture of membranes, oligohydramnios, polyhydramnios, placenta previa, intrauterine growth restriction, gender of offspring, hypertensive disorders of pregnancy, K6 score in T1 and the 14 stressful life events.

^d^ Adjusted for covariates the same as above with 14 stressful life events and without K6 score in T1.

Table 4 shows the results of the parity-stratified analysis. The aOR for PA occurrence was numerically higher (albeit not significantly) in the “death of a child” exposed group relative to the non-exposed group in parous women (aOR 3.28; 95% CI 0.98–10.99). However, the "spouse’s loss of employment" exposed group showed significantly higher odds for PA occurrence than the non-exposed group in parous women (aOR 3.25; 95% CI 1.40–7.56).

**Table 4:** Proportion and adjusted odds ratio of placental abruption occurrence according to parity.

	Placental abruption/n (%)	Adjusted OR	(95% CI)	P-value
Nulliparous (n = 34,773) ^a^				
Not exposed to spouse's loss of employment	152/34,475 (0.4)	1.00	(reference)	
Exposed to spouse's loss of employment	1/298 (0.3)	0.65	(0.09–4.76)	0.67
Parous (n = 46,026) ^b^				
Not exposed to the death of a child	179/45,800 (0.4)	1.00	(reference)	
Exposed to the death of a child	3/226 (1.3)	3.28	(0.98–10.99)	0.055
Not exposed to spouse's loss of employment	176/45,531 (0.4)	1.00	(reference)	
Exposed to spouse's loss of employment	6/495 (1.2)	3.25	(1.40–7.56)	0.006

OR, odds ratio; CI, confidence intervals.

^a^Adjusted for maternal age at delivery, marital status, smoking status, alcohol consumption, use of infertility treatment, maternal education level, pre-pregnancy body mass index, employment status, chronic hypertension, diabetes mellitus, psychiatric disorder, hypothyroidism, uterine deformity, history of uterine fibroids, history of cesarean section, history of spontaneous abortion, history of placental abruption, history of pregnancy-induced hypertension, use of iron supplements during pregnancy, intrauterine infection, premature rupture of membranes, oligohydramnios, polyhydramnios, placenta previa, intrauterine growth restriction, gender of offspring, hypertensive disorders of pregnancy, K6 score in T1 and the other 12 stressful life events; without “illness or injury of a child”.

^b^Adjusted for maternal age at delivery, parity, marital status, smoking status, alcohol consumption, use of infertility treatment, maternal education level, pre-pregnancy body mass index, employment status, chronic hypertension, diabetes mellitus, psychiatric disorder, hypothyroidism, uterine deformity, history of uterine fibroids, history of cesarean section, history of spontaneous abortion, history of stillbirth, history of placental abruption, history of pregnancy-induced hypertension, use of iron supplements during pregnancy, intrauterine infection, premature rupture of membranes, oligohydramnios, polyhydramnios, placenta previa, intrauterine growth restriction, gender of offspring, hypertensive disorders of pregnancy, K6 score in T1 and the other 12 stressful life events.

When compared with the non-PA group, the PA group showed higher proportions of preterm birth, low birth weight, stillbirth, cesarean delivery, neonatal asphyxia, umbilical artery blood acidemia, maternal blood transfusion, maternal ICU hospitalization, and maternal death (S1 Table). PA group also showed the higher proportion of the advanced age, chronic hypertension, hypothyroidism, history of cesarean section, history of placental abruption, history of pregnancy induced hypertension, intrauterine infection, premature rupture of membranes, oligohydramnios, polyhydramnios, placenta previa, intrauterine growth restriction and hypertensive disorders of pregnancy (S2 Table). Excluded participants due to missing data in any variable represent more young, parous, smokers, non-educated, medical complications and obstetric complications occurred (S3 Table).

## Discussion

In this large-scale birth cohort study, we found that exposure to the death of a child one year before or during pregnancy was significantly associated with an increased risk of PA in multigravid women. Additionally, this is the first study that specifically shows an increased risk of PA associated with a spouse’s loss of employment in parous women after adjusting for important confounding factors. However, there were no significant differences detected in the subjective psychological distress score (K6 score) between the PA group and non- PA group for both the T1 and T2 periods.

Our findings that exposure to the death of a child increases the risk of PA corroborate the results of Laszlo et al. [9], which reported that this stressful event was associated with a 54% increase in the odds of PA in a study of 2.8 million normotensive women. That study utilized national statistics to focus on women with at least one living older child one year before pregnancy. As we were not able to obtain that information for our study, our analysis was conducted on multigravid and parous women. As indicated in the baseline characteristics in Table 2, the "death of a child" exposed group had significantly higher proportions of previous spontaneous abortion, stillbirth, and PA. Nevertheless, the relationship between the exposure to death of a child and PA remained statistically significant even after adjusting for these previous events. It is therefore possible that participants had perceived the “death of a child” response in the questionnaire to include a history of miscarriage and stillbirth, and that these were also recognized as the death of a child. In other words, these events may be sufficiently traumatic to increase the risk of developing PA. However, the aOR for the “death of a child” exposed group in parous women was not significant, which may have been the result of insufficient statistical power due to stratification [22]. Although de Paz et al. [10] reported that subjective depressive symptoms and anxiety scores were independently related to an elevated risk of PA, our study showed no difference in K6 scores between the PA group and non-PA group. Additional multivariable analyses did not detect any significant association between K6 score and PA. De Paz et al.[10] was a case-control study, and it is possible that recall bias affected their results. Our study was a prospective study, and indicated that simple subjective psychological distress may not be associated with the risk of PA.

A possible mechanism to explain the effect of stressful life events on PA occurrence is that stress exposure promotes the secretion of stress hormones, which is followed by vasoconstriction, rises in blood pressure [23], and pro-inflammatory [24, 25] and pro-thrombotic [26] changes that result in uteroplacental ischemia [27]. This stress-induced pathology may cause IUGR [6, 7] or pre-eclampsia [8], thereby leading to the occurrence of PA [9, 10]. However, the effects of the death of a child on PA among multigravid women remained significant even after adjusting for IUGR and pre-eclampsia, therefore indicating that women who had been exposed to these events may have experienced more direct effects. While the development of pre-eclampsia and hypertensive disorders of pregnancy are known to be important risk factors for PA [1], 287 (85.7%) of the 335 women with PA in our study did not develop hypertensive disorders of pregnancy.

Another possible mechanism is that sympathetic arousal may have induced PA [28]. Cohen et al. reported a case of PA in a non-medicated pregnant woman with panic disorder [29]. Stressful life events can lead to panic disorders [30], which cause panic attacks and elevate blood pressure [31]. However, our analysis did not allow us to examine panic disorder as a risk factor for PA. Although many panic disorders are known to complicate depressive symptoms [32], our results did not detect any difference in the K6 scores between the PA group and non- PA group. We had performed adjustments for psychiatric disorders, but were unable to identify detailed diagnoses. Further investigations may be needed to clarify the possible influence of each psychiatric disorder such as schizophrenia, depression and panic disorder on PA.

This study has the strengths of being a large-scale prospective cohort study involving recruitment from the Japanese general population [11–13], many of the variables have low risk of recall bias, and adjustments for many important confounding factors. In addition, information on many of these confounding factors and the diagnoses of PA were obtained from medical records. However, the study has several limitations. First, there is the possibility of information bias regarding the stressful life events due to the self-administered nature of the questionnaire and its recall period was set to one year. In addition, the information about the “death of a child” exposure in multigravid women included those who experienced spontaneous abortions or stillbirth, which may lead to an important information bias if the “death of a child” should be limited to the death of a child that has already been born. Nevertheless, our questionnaire was still able to predict the significantly elevated risk for PA. Because the main tools for identifying the risk factors of pregnant women often rely on interview sheets, this self-administered questionnaire may help to reduce labor in the survey process during clinical practice. Second, the K6 score and 12 of the 14 stressful life events in the year before the T2 period were not associated with PA. The death of a child is considered to be a catastrophic stressor [33] and is therefore not surprising as a significant predictor of PA. However, we performed a total of 18 multivariable analyses, thus, the significant associations we observed could be chance findings (type 1 error). If we perform Bonferroni correction, statistical significance should be set at 0.0028 and our results did not show the significant value, however, the consensus about adjustment for the multiple comparison still remains a matter of debate [34] thus, we did not perform Bonferroni correction or other methods in this study. Third, our data on stressful life event exposures did not include detailed timings of their occurrence. Personal perceptions on stress can differ [35], and earlier onset of events can be perceived as being more stressful than later in pregnancy. We were unable to examine the possible effects of the timing of these events on our results. Fourth, there could be an information bias on the prevalence of PA occurrence. According to Matsuda et al. [36], the proportion of PA in Japan was reported as 2461 / 242,715 (1.0%), however, our data showed 0.4% and it was lower than that of the previous PA study. There may be a possibility of an underestimation in PA diagnosis in our data though, the participants of the previous study had the tendency to be biased into severe condition women compared to the Japanese general population because the participated hospitals of their study consisted with many perinatal medical centers. We performed our study targeting the recruitment rate as 50% or more of all eligible mothers [11–13], and confirmed that the JECS participants were approximately similar to the Japanese Vital Statistics [12, 13], so that the proportion of PA in our data would be more similar to the Japanese general population. Fifth, there were some differences in baseline characteristics between the excluded group and the analyzed group (S3 Table). Although the JECS participants were similar to the general population in Japan, some selection bias may exist in the final analyzed sample. Sixth, there could be a possibility of multicollinearity because we used many covariates in the analyses, however, we confirmed that the variance inflation factor (VIF) [37] in each variable was less than five, so that the risk of multicollinearity was not problematic in our analysis. Seventh, there may be the risk of reverse causality, therefore, we examined the data acquisition timing for six persons who experienced both "death of a children" exposure and PA onset. For all six of them, we confirmed that the data about "death of a child" exposure was acquired before the onset of PA. Eighth, the mechanisms underlying these relations are unknown. The K6 score and 12 of the 14 stressful life events were not associated with PA thus, there could be a mechanism that is different from psychological stress, such as biological vulnerability. Ninth, the effect of “spouse’s loss of employment” on PA differs between nulliparous and parous women, and this may be caused by an effect modification. In order to clarify whether this result is really different, we added “parity x spouse’s loss of employment” into full logistic regression model which also includes the measured exposures: all stressful life events and also K6 score (T1) among all women. As a result, no significant association was found in the interaction term. In other words, its mechanism is unclear though, it is considered that “spouses loss of employment” would be a significant risk of PA in parous women.

In conclusion, this study showed the effect of exposure to the death of a child one year before or during pregnancy on PA occurrence in multigravid Japanese women using a large-scale birth cohort study that adjusted for many potentially important confounders. Exposure to a spouse’s loss of employment was also shown to elevate the risk of PA in parous women though, further researches are needed to confirm this relationship. Due to the characteristics of these exposure variables, randomized controlled trials are impractical and the best evidence is dependent on prospective cohort studies. For clinical obstetricians, identification of the death of a child experience as a significant risk factor of PA may help to differentiate the possibility of this condition.

## Supporting information

S1 Table Obstetrical outcomes according to clinical diagnosis of placental abruption. (XLSX)

S2 Table Participant characteristics according to clinical diagnosis of placental abruption. (XLSX)

S3 Table Baseline characteristics comparison of the final analyzed participants and the excluded participants due to missing data in any covariate, outcome variable, or exposure variable. (XLSX)

## Abbreviations

JECS: Japan Environment and Children’s Study
